# A multi-method approach to the molecular diagnosis of overt and borderline 11p15.5 defects underlying Silver–Russell and Beckwith–Wiedemann syndromes

**DOI:** 10.1186/s13148-016-0183-8

**Published:** 2016-03-01

**Authors:** Silvia Russo, Luciano Calzari, Alessandro Mussa, Ester Mainini, Matteo Cassina, Stefania Di Candia, Maurizio Clementi, Sara Guzzetti, Silvia Tabano, Monica Miozzo, Silvia Sirchia, Palma Finelli, Paolo Prontera, Silvia Maitz, Giovanni Sorge, Annalisa Calcagno, Mohamad Maghnie, Maria Teresa Divizia, Daniela Melis, Emanuela Manfredini, Giovanni Battista Ferrero, Vanna Pecile, Lidia Larizza

**Affiliations:** Human Molecular Genetics Laboratory, IRCCS Istituto Auxologico Italiano, Milano, Italy; Department of Pediatric and Public Health Sciences, University of Turin, Torino, Italy; Clinical Genetics Unit, Department of Women’s and Children’s Health, University of Padua, Padova, Italy; Department of Pediatrics, San Raffaele Scientific Institute, Milano, Italy; Division of Pathology - Fondazione IRCCS Ca’ Granda Ospedale Maggiore Policlinico, Department of Pathophysiology and Transplantation, University of Milan, Milano, Italy; Department of Health Sciences, University of Milan, Milano, Italy; Medical Genetics Unit, Department of Surgical and Biomedical Sciences, University of Perugia, Hospital “S. M. della Misericordia”, Perugia, Italy; Clinical Pediatric Genetics Unit, Pediatrics Clinics, MBBM Foundation, S. Gerardo Hospital, Monza, Italy; Department of Pediatrics and Medical Sciences, AO “Policlinico Vittorio Emanuele”, Catania, Italy; Pediatric Endocrine Unit, Department of Pediatrics, IRCCS, Children’s Hospital Giannina Gaslini, Genova, Italy; Department of Medical Genetics, IRCCS, Children’s Hospital Giannina Gaslini, Genova, Italy; Clinical Pediatric Genetics, Department of Pediatrics, University “Federico II”, Napoli, Italy; Medical Genetics Unit, Department of Laboratory Medicine, Niguarda Ca’ Granda Hospital, Milano, Italy; Institute for Maternal and Child Health, Foundation IRCCS Burlo Garofolo Institute, Trieste, Italy

**Keywords:** Beckwith–Wiedemann syndrome, Silver–Russell syndrome, Molecular diagnosis, Mosaic (epi)genetic alterations, Borderline cases, Multi-method approach, MS-MLPA, Pyrosequencing, Southern blot, SNP array

## Abstract

**Background:**

Multiple (epi)genetic defects affecting the expression of the imprinted genes within the 11p15.5 chromosomal region underlie Silver–Russell (SRS) and Beckwith–Wiedemann (BWS) syndromes. The molecular diagnosis of these opposite growth disorders requires a multi-approach flowchart to disclose known primary and secondary (epi)genetic alterations; however, up to 20 and 30 % of clinically diagnosed BWS and SRS cases remain without molecular diagnosis. The complex structure of the 11p15 region with variable CpG methylation and low-rate mosaicism may account for missed diagnoses. Here, we demonstrate the relevance of complementary techniques for the assessment of different CpGs and the importance of testing multiple tissues to increase the SRS and BWS detection rate.

**Results:**

Molecular testing of 147 and 450 clinically diagnosed SRS and BWS cases provided diagnosis in 34 SRS and 185 BWS patients, with 9 SRS and 21 BWS cases remaining undiagnosed and herein referred to as “borderline.” A flowchart including complementary techniques and, when applicable, the analysis of buccal swabs, allowed confirmation of the molecular diagnosis in all borderline cases. Comparison of methylation levels by methylation-specific multiplex ligation-dependent probe amplification (MS-MLPA) in borderline and control cases defined an interval of *H19/IGF2:*IG-DMR loss of methylation that was distinct between “easy to diagnose” and “borderline” cases, which were characterized by values ≤mean −3 standard deviations (SDs) compared to controls. Values ≥mean +1 SD at *H19/IGF2:* IG-DMR were assigned to borderline hypermethylated BWS cases and those ≤mean −2 SD at *KCNQ1OT1:* TSS-DMR to hypomethylated BWS cases; these were supported by quantitative pyrosequencing or Southern blot analysis. Six BWS cases suspected to carry mosaic paternal uniparental disomy of chromosome 11 were confirmed by SNP array, which detected mosaicism till 10 %. Regarding the clinical presentation, borderline SRS were representative of the syndromic phenotype, with exception of one patient, whereas BWS cases showed low frequency of the most common features except hemihyperplasia.

**Conclusions:**

A conclusive molecular diagnosis was reached in borderline methylation cases, increasing the detection rate by 6 % for SRS and 5 % for BWS cases. The introduction of complementary techniques and additional tissue analyses into routine diagnostic work-up should facilitate the identification of cases undiagnosed because of mosaicism, a distinctive feature of epigenetic disorders.

**Electronic supplementary material:**

The online version of this article (doi:10.1186/s13148-016-0183-8) contains supplementary material, which is available to authorized users.

## Background

The 11p15.5 chromosomal region contains two clusters of imprinted genes, the monoallelic expression of which is independently regulated by cis-acting differentially methylated regions (DMRs) or imprinting control centers (ICR), ICR1 (alias *H19/IGF2:*IG-DMR) and ICR2 (alias *KCNQ1OT1*:TSS-DMR), corresponding to sequences methylated on one parental allele [1]. *H19/IGF2:*IG-DMR, which is located upstream of the *H19* promoter, controls the telomeric cluster containing the *IGF2* and *H19* genes, which are expressed on the paternal and maternal chromosomes, respectively. *KCNQ1OT1*:TSS-DMR, lying within an intron of the *KCNQ1* gene, regulates the centromeric cluster containing the *CDKN1C* and *KCNQ1* genes, which are expressed by alleles of maternal origin, and the *KCNQ1* antisense transcript *KCNQ1OT1*, which is expressed on the paternal allele [2–4]. Multiple genetic and epigenetic defects lead to the congenital Silver–Russell (SRS) and Beckwith–Wiedemann (BWS) syndromes, which are characterized by opposite growth disorder phenotypes and imbalances in the expression levels of the resident imprinted genes. The variety of (epi)genetic alterations and the complex structural organization of the 11p15.5 region account for the difficult molecular diagnostics of these clinically and genetically heterogeneous disorders [5, 6]. Currently, loss of methylation (LoM) of the paternal ICR1-*H19* locus is present in >50 % of SRS patients, whereas gain of methylation (GoM) of the maternal ICR1-*H19* is observed in 5 % of BWS patients and has recently been suggested to be more common than previously thought [7]. More than 50 % of BWS patients display LoM at the maternal *KCNQ1OT1*:TSS-DMR, leading to biallelic expression of the *KCNQ1OT1* long non-coding transcript and silencing of the active maternal alleles. More than 20 % of BWS patients carry mosaic paternal uniparental disomy of chromosome 11 (upd(11)pat), with a great variability in the level and extent of isodisomy. After adding 5–10 % of BWS patients positive for loss-of-function *CDKN1C* mutations and a small (1–2 %) fraction of 11p familial or de novo rearrangements causing duplication of the paternal chromosome, up to 20 % of clinically diagnosed BWS cases remain without a molecular diagnosis [8, 9]. A similar situation occurs for SRS, where 10 % of the patients carry maternal upd7 and a tiny fraction (up to 4 %), slightly higher than that for BWS, carry structural rearrangements of the maternal chromosome 11p [5, 10]. In parallel, familial forms of SRS bear gain of function mutations of the PCNA domain of *CDKN1C* [11], further confirming that opposed functional mutations underlie the opposite phenotypes. Because several primary or secondary epigenetic mechanisms associated with BWS and SRS occur at a low mosaic level, complementary techniques interrogating different CpGs of the target genes and their flanking regions, including quantitative methylation-specific pyrosequencing [12, 13] and in some cases analysis of a tissue other than blood [14], are recommended to confirm uncertain results. The common mosaic paternal UPD11 underlying BWS usually requires parents for proband microsatellite analysis; however, SNP array might highlight overlooked low mosaic upd [15], in addition to providing fine mapping of the recombination breakpoints [16]. Similarly, detection of the rare but observable structural rearrangements or copy number variations causing subtle imbalances of the subtelomeric 11p15.5 region requires the integrated application of multicolor fluorescence in situ hybridization (FISH), high-resolution array comparative genomic hybridization (CGH), or SNP array [17–20]. Cis- and trans-acting factors responsible for complex interactions between the 11p15.5 imprinted genes have been identified in both BWS and SRS [7, 21, 22]; however, they are not included in the current diagnostic flowchart as no precise information is available on the fraction of cases accounted for by these mechanisms. Although this point is being addressed by ongoing research, diagnostic laboratories must combine all the indicated approaches to obtain the highest possible detection rate (>80 %) of (epi)genetic alterations in SRS and BWS.

In the course of our diagnostic activity on 450 patients within the BWS spectrum and 147 SRS, including 130 likely and 17 unlikely SRS [23], we encountered a set of 21 BWS and 9 SRS cases, comprehensive of an unlikely case, in which a conclusive molecular diagnosis was lacking upon a standard (epi)genetic test. These cases are herein referred to as “borderline” and are the main subjects of the present study. They exemplify the difficulties in confirming the clinical diagnosis and underscore the need to combine Southern blotting (SB) with methylation-specific multiplex ligation-dependent probe amplification (MS-MLPA) and bisulphite pyrosequencing and microsatellite segregation analysis with SNP array using patients’ blood and, when possible, additional tissues, to obtain an unequivocal molecular diagnosis. Identification of low-level mosaic upd(11)pat, which implies a high cancer risk in BWS [24] and in its mildest phenotypic end (i.e., pure isolated hemihyperplasia (IH)) [25] has a strong translational impact, as it allows proper oncological surveillance and patient management.

## Results

**Fig. 1:**
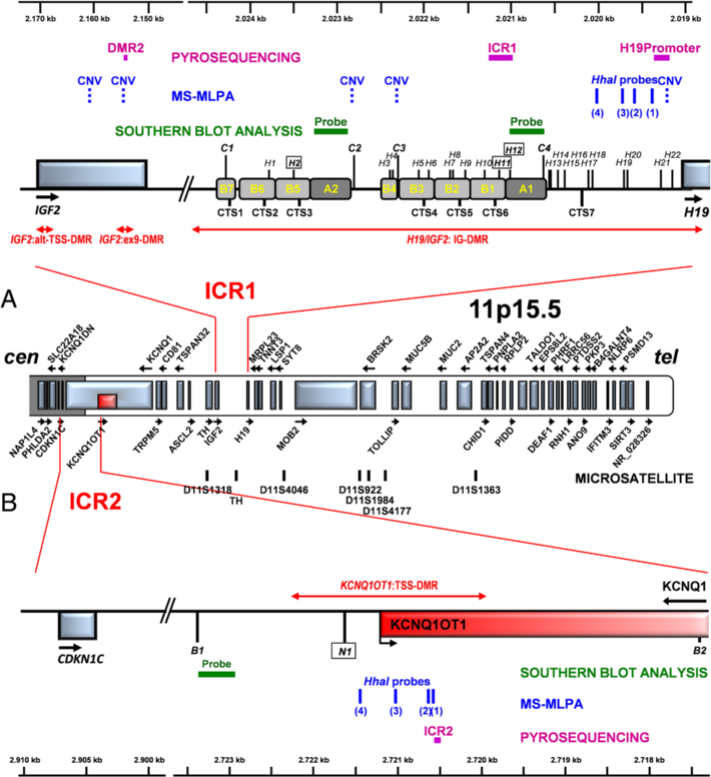
Schematic of the 11p15 imprinted region. ICR1 comprising *H19/IGF2*:IG-DMR, organized into the two CTS1-3 and CTS 4–6 clusters, plus the proximal CTS7, *IGF2*:ex9-DMR, and *IGF2*:alt-TSS-DMR (**a**) and ICR2 comprising *KCNQ1OT1*:TSS-DMR (**b**) are depicted. *Red lines ending with arrowheads* point to the DMRs. The *HpaII* sites, widely distributed across *H19/IGF2*:IG-DMR, are indicated by the *vertical H bars*; *letters in the boxes* represent CpG sites analyzed by SB. The *Csp6I* (**a**), *BamHI*, and *NotI* (**b**) sites are indicated by the *vertical C*, *B*, and *N bars*, respectively. *Green horizontal bars* indicate the probes for SB, *blue vertical* bars indicate the MLPA probes (*dashed* for CNVs and *solid* for methylation status), and *solid purple bars* indicate the pyrosequencing target regions [12]. The *numbers in brackets* of MLPA methylation probes refer to those indicated with the relative code in Fig. 2f, g

Figure 1 shows a schematic representation of the complex architecture of the 11p15.5 imprinted region, including *H19/IGF2:*IG-DMR, with its cluster organization (CTS1-CTS7) and *IGF2*:ex9-DMR, *IGF2*:alt-TSS-DMR (a) and *KCNQ1OT1*:TSS-DMR (b). As shown for both imprinted domains, the DMRs targeted by the probes used in SB, MS-MLPA, and pyrosequencing primers [8, 12, 26, 27] are different, indicating that the available tools are not redundant, but rather complementary, for investigating the 11p15.5 methylation profile.

### Definition of standard parameters for MS-MLPA

MS-MLPA is currently the most rapid and robust technique to assess methylation. Therefore, before combining it with other techniques, we established the proper range of values in the normal population. Control DNA obtained from blood samples of 50 normal individuals was used to calculate for each probe the mean value (±1–3 standard deviation (SD) points). As shown in Additional file 1: Table S1, the mean values varied according to the probes and the range from the lowest to the maximum values. All samples fell within the 2 SD range for both *H19/IGF2*:IG-DMR and *KCNQ1OT1*:TSS-DMR, allowing definition of the range in healthy individuals.

### Complementary use of SB, MS-MLPA, and pyrosequencing

Various 11p15.5 targeted techniques, such as Southern blot (SB), MS-MLPA, and pyrosequencing, were combined to compare their sensitivity in mosaicism detection. Despite the multi-disciplinary technical approach, additional tissues were analyzed in borderline cases to obtain an unequivocal molecular diagnosis.

The clinical diagnosis of SRS was confirmed by genetic testing in 43 of 147 patients (29 %) with suspected SRS. Of these, seven patients carried upd(7)mat (4.8 %), one of which scored as unlikely SRS, whereas paternal *H19* hypomethylation was detected in the remaining 36 (24.4 %) cases. Mosaic *H19/IGF2:*IG-DMR LoM was definitely detected in 27 cases by SB and those samples were used for the set up of MS-MLPA (since it was available) and pyrosequencing tests. Concerning the remaining nine SRS, (eight clinically likely and one unlikely) belong to the borderline cases focus of this study. Additional file 2: Table S2 shows how many SRS patients achieved a molecular diagnosis by single and/or combined SB/MS-MLPA/pyrosequencing techniques.

The clinical diagnosis of BWS was confirmed genetically in 206 of 450 patients, accounting for a detection rate of 46 %. The epigenetic defects were detected by the use of a single technique with definite values in 120 (27 %) mosaic maternal LoM at *KCNQ1OT1*:TSS-DMR patients, in nine (2 %) mosaic maternal GoM at *H19/IGF2*:IG-DMR and in 46 cases of mosaic paternal UPD including seven cases with additional altered copy number variants (CNVs) indicative of a microdeletion/duplication [18, 28], [unpublished]; some of these cases were used to validate alternative techniques as detailed in Additional file 2: Table S2. Despite the SB has been now overlooked by more recent techniques, in the past, it was the only available test.

Thanks to the complementary technical approaches used the diagnosis could be also confirmed in 9 SRS and in 21 BWS cases with borderline methylation levels. The availability of buccal smears was fundamental in sustaining the molecular diagnosis of 8 BWS and 3 SRS cases within the borderline subset and definitely allowed to sort out from the positive set a couple of cases.

### Low-level mosaic epimutations

**Fig. 2:**
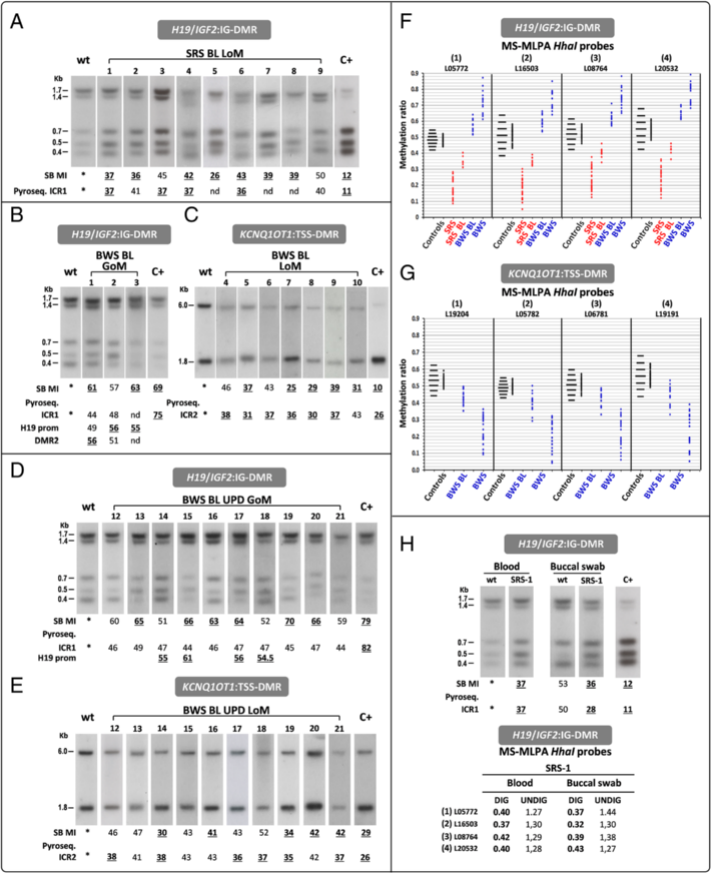
(Epi)genetic alterations of SRS and BWS borderline (BL) cases assessed by SB, pyrosequencing and MS-MLPA. **a** Autoradiograms of borderline SRS patients showing a subtle increase in the density of the unmethylated band for *H19/IGF2*:IG-DMR. In the borderline BWS patients, autoradiograms show a slight increase in density at the methylated alleles at the *H19/IGF2*:IG-DMR (**b**, **d**) and at the unmethylated allele at the *KCNQ1OT1*:TSS-DMR (**c**, **e**). Under each southern lane, methylation indexes (MI) obtained by densitometry quantification are shown and pyrosequencing methylation percentages are provided for the following probes: ICR1 (**a**); ICR1, *H19* promoter and DMR2 (**b**); ICR2 (**c**, **e**); and ICR1 and *H19* promoter (**d**): see Additional file 1: Table S1 and the “Methods” sections for the normal ranges and the thresholds accordingly set up. Aberrant methylation values are shown by *bold underlined* numbers. The diagrams in **f** and **g** show the distribution of MS-MLPA methylation values obtained with the *HhaI* probes in 50 normal individuals (Additional file 1: Table S1). The *horizontal black bars* (left to the control methylation distributions) define the interval between the mean value and ±1 to 3 SDs. All values at *H19/IGF2*:IG-DMR and *KCNQ1OT1*:TSS-DMR are depicted by *dots* (*red* for SRS and *blue* for BWS). Hypomethylated (SRS), borderline hypomethylated (SRS BL), 15 representative hypermethylated (BWS), and all borderline hypermethylated (BWS BL) are shown in **f**, while borderline hypomethylated (BWS BL) and 26 representative hypomethylated (BWS) are shown in **g**. See Additional file 3: Table S3 for the methylation values of all borderline cases. Due to the limited availability of DNA, BWS borderline case 11 was not included in the figure as investigated only by pyrosequencing and MS-MLPA (see Additional file 3: Table S3). **h** The results for borderline SRS-1 obtained by SB, pyrosequencing, and MS-MLPA on blood and buccal swab DNA. MS-MLPA CNVs are shown, indicating a small sized gain at locus *H19/IGF2*:IG-DMR

The challenge for molecular diagnosis raised by low-level mosaic epimutations can be addressed by using complementary techniques and multiple tissues. Figure 2 provides representative examples of low-level mosaic epimutations in SRS and BWS. The autoradiograms of the cases with borderline *H19/IGF2*:IG-DMR and *KCNQ1OT1*:TSS-DMR band patterns are shown in Fig. 2a–e. The respective SB densitometric methylation indexes and methylation values obtained by pyrosequencing in 6 of 9 borderline SRS cases and 20 of 21 borderline BWS cases are also shown. The MS-MLPA methylation ratio of all SRS/BWS samples with borderline methylation values are provided in Additional file 3: Table S3. Figure 2f, g displays graphs summarizing the distribution of the values obtained by the MS-MLPA *HhaI* probes for *H19/IGF2*:IG-DMR and *KCNQ1OT1*:TSS-DMR in samples with different degrees of hypo/hypermethylation, including the borderline SRS and BWS cases. The methylation values in the control cohort were distributed within mean values ±2 SD points. The four *HhaI* MLPA probes in *H19/IGF2*:IG-DMR hypomethylated SRS cases had almost all values ≤mean −3 SDs (Fig. 2f). MS-MLPA showed in the borderline cohort values slightly below the cut-off, which were supported by SB (see, i.e., cases 1 and 2) and by pyrosequencing (cases 3 and 6) (Fig. 2a). The mean values among the four probes of MS-MLPA (Additional file 3: Table S3) may give a misleading result (i.e., BWS cases 10 and 13) so we always considered the single values for each probe. The detection of at least two probes lying outside the interval of normal samples indicates possible epimutations at a low mosaic rate and needs to be further validated. On the other hand, the methylation ratios in *H19/IGF2*:IG-DMR hypermethylated borderline BWS cases partially overlapped with those of the normal population for some of the probes; accordingly, cases with values ≥mean +1 SD were considered to be hypermethylated. The three patients showing GoM at *H19/IGF2*:IG-DMR at borderline values by MS-MLPA were supported by SB (cases 1 and 3) and by pyrosequencing (case 1 at DMR2 and cases 2 and 3 at *H19* prom) (Fig. 2b–e).

Of the eight patients with *KCNQ1OT1*:TSS-DMR LoM with values at the cut-off by MS-MLPA, case 10 was only supported by SB, cases 4 and 6 only by pyrosequencing and cases 5, 7, 8, and 9 by both SB and pyrosequencing (data reported in Additional file 3: Table S3) (Fig. 2c).

Whenever possible, cases close to the cut-off by the three techniques (SRS-1, SRS-2, and SRS-9 and BWS-1, BWS-2, BWS-6, BWS-7, BWS-11, BWS-15, BWS-16, and BWS-17) were resolved by analyzing mouth swabs, which provided clear-cut results in some cases. In patient SRS-1 SB, MS-MLPA and pyrosequencing consistently detected slight hypomethylation associated with copy number gain, which is suggestive of microduplication (Fig. 2h). The *H19* methylation ratio was lower in the buccal swab test than in the blood test, confirming the occurrence of the epimutation.

### Low-level mosaic upd(11)pat

Out of 46 BWS patients with both *KCNQ1OT1*:TSS-DMR LoM and *H19/IGF2*:IG-DMR GoM, 42 were confirmed to be mosaic upd(11)pat by 11p15.5 microsatellite analysis, whereas four carried chromosomal rearrangements detected by molecular cytogenetic techniques [18] (unpublished). Slight epimutations were suspected in ten additional cases, as suggested by borderline ratios between the paternal and the maternal alleles in the microsatellite analysis [29], thus hampering a conclusive molecular diagnosis. These cases showed the lowest degree of mosaicism, revealing uncertain results with each technique. Microsatellite ratios helped us to suspect the diagnosis, but the conclusive diagnosis was taken after the SNP array test. Additional file 3: Table S3 highlights the controversial results obtained by the different techniques.

**Fig. 3:**
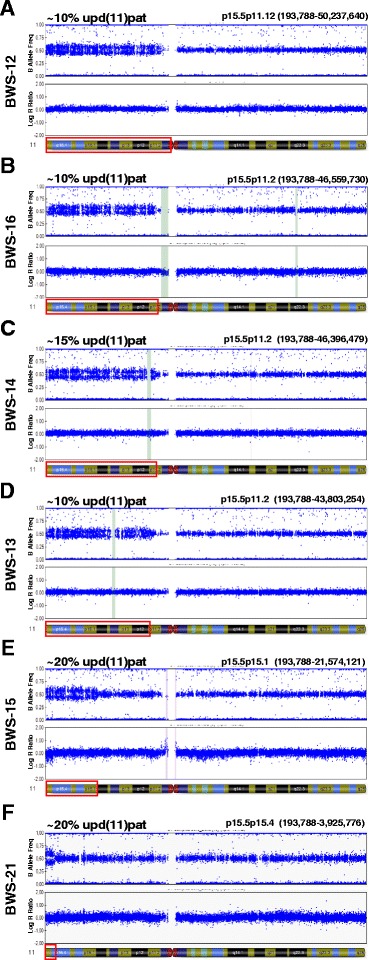
SNP array Bead Studio output of selected BWS borderline cases. All the indicated BWS patients, “suspected” by microsatellite 11p15 analysis, are confirmed to carry a mosaic segmental paternal uniparental disomy (upd(11)pat). Patients are listed from top to bottom according to decreasing extent of the mosaic isodisomy (*framed in red* in the ideogram below each profile). The percentage of upd(11)pat in the DNA from blood cells, calculated from the array data as described (see the “Methods” section), ranges from 20 % (BWS-15, BWS-21) to 15 % (BWS-14) down to 10 % (BWS-12, BWS-13, and BWS-16)

To exemplify the doubtful cases, cases 13 and 16 would have been missed by MS-MLPA, cases 12 and 18 would have been fully missed by SB, and cases 13, 16, and 20 by pyrosequencing (Fig. 2d, e). In six of these cases, SNP array confirmed the low mosaic upd(11)pat rate. As shown by the profiles in Fig. 3, the extent of segmental UPD was highly variable, ranging from the entire arm of chromosome 11p to only the distal 11p15.5–p15.4 cytobands. The rate of mosaicism in blood samples was 10 % in half of the tested patients and from 15 to 20 % in the remaining three cases. The clinical phenotype of the six cases solved by SNP array was consistent with pure IH in BWS-16 and BWS-21 and IH plus one/two additional signs of the BWS spectrum in BWS-12, BWS-13, BWS-14, and BWS-15. Re-evaluation of the MS-MLPA values and microsatellite peaks of the array-SNP-solved cases supported the diagnosis of low-level upd in the remaining four borderline cases.

**Fig. 4:**
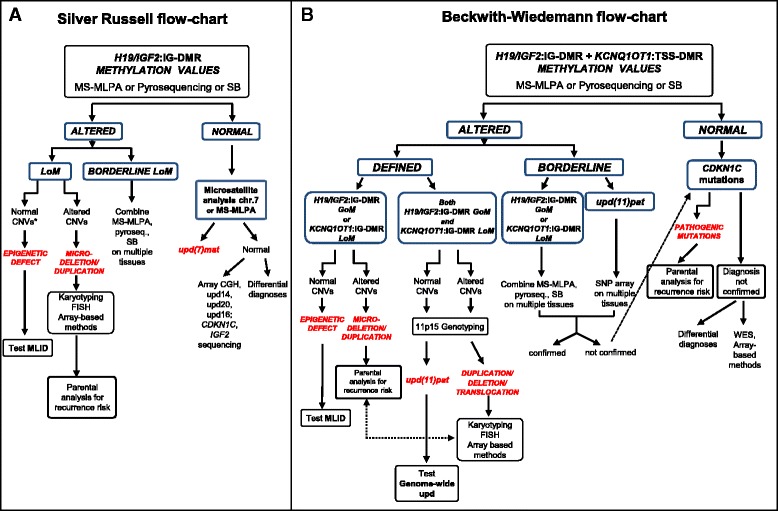
Flowcharts for molecular diagnosis of SRS (**a**) and BWS (**b**) syndromes. **a**. The 11p15.5 methylation test targeted to *H19/IGF2*:IG-DMR is the first assay and is currently performed by MS-MLPA or pyrosequencing or Southern blot. In cases with borderline methylation values, two or more combined techniques are useful and if applicable multiple tissues should be tested. Evidence of LoM with normal CNVs confirms the epigenetic defect, while LoM with altered CNVs suggests a microdeletion/duplication. These cases need to be defined by karyotyping, FISH, or array-based genomic methods which should be extended to probands’ parents in order to define the recurrence risk. Further proceeding in cases with the epigenetic defect is represented by the MLID test. Cases with normal 11p15.5 methylation levels are tested by chromosome 7 MS-MLPA and/or microsatellite analysis to evidence maternal upd(7)mat and possible epimutations. The negative cases are either processed by array-based genomic methods to detect upd at multiple chromosomal regions or sequenced for *CDKN1C* and *IGF2* or addressed to differential diagnoses. **b** The 11p15.5 methylation test targeted to both *H19/IGF2*:IG-DMR and *KCNQ1OT1*:TSS-DMR is the preliminary assay, and alternative options are recommended according to altered (positive) or borderline results. Microsatellite analysis defines upd, while aberrant CNVs raise the suspicion of unbalanced translocation/duplication, which should be verified by karyotyping/FISH/array-based methods and, if confirmed, recommend parental analysis. Further proceedings are represented by MLID test in case of epigenetic defect and genome-wide upd test in case of mosaic paternal upd. Borderline values on a single tissue can be ascertained by the analysis of an additional tissue and techniques, also including SNP array, that may better explore the region. On negative cases, *CDKN1C* mutations are then searched to complete the BWS molecular testing. Should the clinical diagnosis not be confirmed, differential diagnoses have to be taken into account and whole exome deep sequencing and array-based methods should be considered

The SRS and BWS flowcharts outlined in Fig. 4a, b summarize the itinerary of the molecular diagnostic work-up which led to molecular diagnosis in our clinically evaluated SRS and BWS patients, also including low levels epimutated and upd(11)pat cases. We suggest to confirm/rule out the molecular diagnosis of borderline cases by different techniques and, when possible, by testing multiple tissues. Chromosome 7 upd should follow on the 11p epigenetically negative SRS cases, while *CDKN1C* sequencing is recommended in the negative to methylation test BWS cases. Further proceedings, to achieve the molecular diagnosis and match it to the clinical phenotype are outlined too, even if they have not yet entered in the current diagnostics.

### Clinical features of borderline patients

Tables 1 and 2 provide the clinical data of borderline SRS patients (*n* = 9, aged 8 months to 13 years) and BWS patients (*n* = 21, 11 mosaic epimutated and 10 mosaic upd(11)pat cases, aged 10 days to 14.5 years). The latter group included the six BWS patients in which the upd(11)pat diagnosis needed to be confirmed by SNP array.

**Table 1:** Clinical features of borderline Silver–Russell cases

Cases	1	2	3	4	5	6	7	8	9
Sex	M	M	F	F	M	M	M	M	F
Age at last evaluation	6 y	2.9 y	4.1 y	8 y	2.5 y	13 y	1 y	2.6 y	8 m
ART	−	−	+	−	+	−	−	−	−
IUGR	+	+	+	+	+	−	+	+	−
Oligohydramnios	nr	−	−	−	−	−	−	−	−
Weeks at delivery	37 + 4	34 + 6	35 + 4	40	34	42	37 + 2	37	38
Birth weight (g %)	1860 <3 %	1670 <3 %	1970 <10 %	2380	1200 <3 %	nr	2000 3 %	2250 5 %	2300 <5 %
Birth length (cm %)	42 <3 %	41 <3 %	41 <3 %	49	39 <3 %	nr	43	47 5 %	43 <3 %
CC (cm %)	33 10 %	32	33.5 75 %	33	31 50 %	nr	nr	33 10 %	nr
Weight (%) at last evaluation	<5 %	3 %	<3 %	<3 %	<3 %	50−75 %	<3 %	<5 %	<3 %
Height (%) at last evaluation	<5 %	10 %	10 %	25 %	<3 %	50 %	3 %	<5 %	<3 %
CC (cm %) at last evaluation	52 cm	50 cm	50 %	75 %	25 %	50 %	50 %	75−90 %	nr
Feeding difficulties	−	+	−	−	−	−	−	−	+
GER	−	−	+	−	−	−	−	−	−
Triangular face	+	−	+	−	+	−	+	+	+
Prominent forehead	+	+	+	+	+	−	+	+	+
Small chin	+	−	+	−	+	−	−	−	+
Downslanting corner of mouth	−	−	+	−	−	−	−	−	+
Thin lips	+	−	+	−	+	+	−	−	+
Ear anomalies	+	+	+	+	−	+	−	+	+
Asymmetry	−	+	+	+	+	+^a^	+	−	+
Clinodactyly V	+	−	−	−	−	−	+	−	−
Syndactyly	+	−	−	−	−	−	−	−	+
Cafè au lait spots	−	+	+	−	−	−	nr	−	nr
Muscular hypotrophy/hypotonia	−	−	+	−	+	−	−	nr	+
Hypospadia	−	+	−	−	+	−	−	−	−
Psychomotor delay	−	−	−	−	+	−	−	+	+
Malformations and pediatric problems	Transitory neonatal hypocalcemia	Peritoneal simple mesothelial cyst	–	–	Epicantus, cryptorchidism, preputial abnormality	Bilateral epicantus	–	–	–
Other	Unique umbilical artery GH deficit	–	Minor MAF	–	Paravertebral neuroblastoma	–	–	–	–

*y* years, *m* months, *nr* not reported, *g* grams, *CC* cranial circumference, *GER* gastroesophageal reflux, *MAF* fetal active movements

^a^Face, ears, upper and lower limbs, tonsillae, kidney

**Table 2:** Clinical features of borderline BWS cases

	*H19*/*IGF2*:IG−DMR			*KCNQ1OT1*:TSS−DMR								upd(11)pat									
	GoM			LoM																	
Cases	1	2	3	4	5	6	7	8	9	10	11	12	13	14	15	16	17	18	19	20	21
Sex	F	F	F	F	F	F	F	M	F	M	F	M	M	M	M	M	F	F	F	M	F
Age at last evaluation	5.4 y	9 m	10 y	7 y	3.8 y	6.5 y	1.5 y	10 d	1 y	13 m	5 m	2.5 y	5.6 y	2.5 y	4 y	9 y	6 y	1 y	3.5 y	14.5 y	8 m
Miscarriages	+	−	−	−	−	−	−	−	−	−	−	−	−	−	nr	nr	−	−	−	nr	+
Polyhydramnios	−	−	−	−	+	−	−	−	−	−	−	−	−	−	nr	nr	−	−	−	−	−
ART	−	−	−	−	+	−	−	−	+	−	−	−	nr	−	nr	nr	−	−	−	−	−
Monozygous twin	−	−	−	−	−	+	+	−	−	−	−	−	−	−	−	−	−	−	−	−	−
Weeks at delivery	41	37	34	38	39 + 4	36	30	40 + 3	40 + 5	39	41	36 + 4	39	nr	nr	39	40	38	41	40	40
Birth weight (g)	3790	4030	2280	2095	4085	2060	770	4590	3880	3150	4430	3130	4275	nr	nr	3910	2815	+	3650	4140	−
Birth length (cm)	52	53	nr	nr	53	42	31	52	52	51	54	48	49	nr	nr	53	48	nr	51	nr	nr
CC (cm)	nr	38	nr	nr	36	33	23	35	33.5	35	38	nr	35	nr	nr	36	nr	nr	34	nr	nr
Postnatalovergrowth	−	+	−	−	+	−	−	+	−	+	nr	−	−	−	−	−	−	+	−	−	−
Hypoglycemia	−	−	+	−	+	−	+	−	−	−	+	−	−	−	−	−	−	−	+	+	−
Macroglossia	−	−	−	−	−	−	+	−	+	−	+	−	−	+	−	−	−	±	+	+	−
Facial dysmorphology	−	−	−	−	−	−	+	−	+	−	+	−	−	+	−	−	−	−	+	−	−
Ear pits/creases	+	+	−	−	−	−	−	+	−	−	−	−	−	−	+	−	−	−	−	+	−
Hemihyperplasia	+	+	+	+	+	+	−	+	+	+	−	+	+	+	+	+	+	+	−	+	+
Facial asymmetry	−	−	+	+	−	+	−	−	−	−	−	−	+	−	+	−	−	+	−	−	−
Exomphalos	−	−	−	−	−	−	+	−	−	−	−	−	−	−	−	−	−	−	+	−	−
Rectum diastasis	−	−	−	+	−	−	+	+	+	−	−	−	+	−	−	−	−	−	−	−	−
Umbilical hernia	−	−	−	−	−	−	−	+	−	−	+	+	−	−	+	−	−	+	+	−	−
Organomegaly	−	−	−	−	−	−	−	−	−	−	−	−	−	Liver	−	−	−	−	−	−	−
Naevus flammeous	−	−	−	−	−	+	−	+	−	−	−	−	−	−	−	−	−	−	+	−	−
Wilms’ tumor/onset age	4 y	−	2.5 y	−	−	−	−	−	−	−	−	−	−	−	−	−	−	−	−	−	−
Convulsions	−	−	−	−	−	−	+^a^	−	−	−	−	−	−	−	−	−	−	−	−	−	−
Psychomotor delay	−	−	+	−	−	+	+	−	−	−	−	−	−	−	−	−	−	−	−	−	−
Other	−	−	IUGR cleft palate, dilated submeningial cavities	−	−	VSD, PFO	Hypo-thyroidism		−	−	−	−	−	PAS	−	Renal hypoplasia	Telarca	Thyroid nodule	−	−	Adrenal nodule

*y* years, *m* months, *d* days, *nr* not reported, *ART* assisted reproductive technologies, *g* grams, *CC* cranial circumference, *VSD* (perimembranous, subaortic) ventricular septal defect, *PFO* patent foramen ovale, *PAS* pulmonary arterial stenosis

^a^Paroxysmal tonic upgaze

**Fig. 5:**
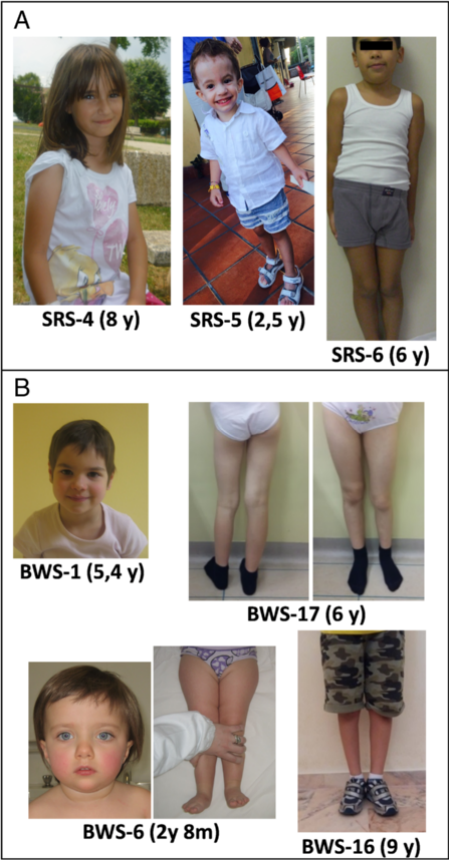
Variable clinical features of SRS and BWS patients with low mosaic methylation levels or upd. **a** Face and full body of SRS-4 at age 8 years, SRS-5 at 2.5 years, and SRS-6 at 6 years of age. No overt facial dysmorphisms are visible in SRS-4; frontal bossing is marked in SRS-5, while SRS-6 shows only hemihypoplasia of the left lower limb. **b** Frontal face of BWS-1 at 5.4 years of age, showing mild but typical features, BWS-6 at 2 years and 8 months, showing no facial dysmorphisms and only pronounced hemihyperplasia of the lower right limb, BWS-16 at 9 years and BWS-17 at 6 years with visible hemihyperplasia of lower left and right limb, respectively

All our borderline SRS patients presented with the characteristic of likely SRS, according to Netchine-Harbison criteria [23], with the exception of SRS-6, who did not show any SRS sign except body asymmetry. The latter was observed in 7/9 (77 %) cases (Table 1). Regarding common BWS clinical signs, birth overgrowth was present in 6/19 (31 %) patients, postnatal overgrowth in 5/20 (25 %), macroglossia in 7/21 (33 %), hypoglycemia in 6/21 (28 %), and omphalocele in 2 cases (<1 %). By contrast, hemihyperplasia was observed in 18/21 (86 %) probands and was isolated in three patients (BWS-16, BWS-17, and BWS-21) and associated with only a minor BWS sign in six patients (BWS-1, BWS-4, BWS-6, BWS-12, BWS-14, and BWS-15). Methylation anomalies at the two ICRs and upd(11)pat were the causative mechanisms of these borderline cases, mostly lying at the mildest end of the wide BWS spectrum. Consistent with these observations, the facial dysmorphisms typical of SRS and BWS were only detected in a few cases and were absent in others, as shown in Fig. 5. In line with the variable clinical phenotype of the borderline BWS patients, unusual findings included intrauterine growth restriction (IUGR), cleft palate, and dilated submeningial cavities in BWS-3, and renal hypoplasia in BWS-16. Of 21 BWS borderline patients, two belonged to monozygotic twin pairs. One of the borderline SRS patients developed a paravertebral neuroblastoma at age 2.5 years, and two BWS patients developed Wilms tumor at 2.5 and 4 years, respectively. Two SRS and two BWS patients were conceived by artificial reproductive techniques.

## Discussion

The objective of the present work was to integrate complementary techniques to improve the identification of SRS and BWS patients with 11p15 abnormalities whose diagnosis may have been missed using standard genetic tests. Although pyrosequencing of *H19/IGF2*:IG-DMR and *KCNQ1OT1*:TSS-DMR [12, 13] and SNP arrays [15, 16] are effective techniques for the evaluation of low-level mosaic epimutations and UPD, neither technique is per se sufficient to disclose the entire spectrum of alterations. Combining these techniques is important, especially in cases in which methylation levels are near the normal threshold of control individuals. In addition, the analysis of tissues other than blood is important for detecting IH patients who, despite showing a mild phenotype, would benefit from accurate genetic counseling and specific cancer surveillance programs given their high risk of developing embryogenetic tumors [25]. In order to detect hidden mosaicism in BWS/SRS, collection of buccal swabs, urine, or cells of mesenchymal origin in conjunction with surgery should be implemented in the practical molecular work-up being facilitated by the common need for interventions in childhood to correct macroglossia, abdominal wall defects, or severe limb asymmetry.

We believe that two relevant aspects are at the roots of the complexity of BWS and SRS molecular diagnosis. First, the variable degree of methylation at the different CpGs within the *H19/IGF2*:IG-DMR region, which was reported recently in SRS [30] and previously in BWS [31], requires that the tools of molecular diagnosis extensively interrogate the differentially methylated CpGs in order to assure the maximum coverage. This is particularly true when a single CpG suggests deregulated methylation. Azzi [30] and Cerrato’s [31] studies found that CTS1 and CTS7 tend to be correctly methylated in the presence of deregulated flanking CpGs. In the present study, we analyzed methylation patterns at the *H19* promoter, A1 and B1 repeats (CTS6) by at least two approaches, while at B5 repeat (CTS3) and *IGF2* by SB and pyrosequencing, respectively (see Fig. 1). In agreement with Azzi et al. [30], we observed that methylation recorded at *H19* promoter and CTS3 gave overlapping results, while values at CTS6 may be normally methylated in patients, suggesting that some sites may be less representative than others. To provide a key for the interpretation of borderline molecular results obtained by MS-MLPA, which is one of the most widely applied tools in BWS/SRS diagnostic laboratories, we first compared the range of methylation levels in controls to those in affected individuals (Additional file 1: Table S1 and Fig. 3f, g). In patients with SRS, we defined a *H19* hypomethylation interval that allowed distinction between “easy to diagnose,” with values ≤mean −4 SD and “borderline” values, with values between mean −3 SD and mean −4 SD. In SRS, we succeeded in defining a “disease range” of *H19* LoM, i.e., values that were never observed in normal controls, although with a narrow threshold [23].

Average methylation levels should be interpreted critically by assessing the values for each probe, as a patient may show normal indexes for one probe and aberrant ones for another probe. In these cases, the use of other techniques or expanding the analysis to tissues other than blood becomes necessary [31]. The inclusion of different methods allows zooming in on the entire region to be investigated, which enhances the diagnostic ability in borderline cases. The second diagnostic pitfall is the occurrence of low-grade mosaicism in BWS carriers of upd(11)pat. These patients are at risk of misdiagnosis when only methylation values are analyzed, as more than one CpG may fall within the normal range despite the use of different technical approaches. When the mean MS-MLPA blood values fall within 1 SD, detection of upd is recommended using microsatellite segregation or, even better, SNP array, as the latter method covers a more extensive region and is therefore more sensitive. SNP array validation in some cases and buccal swab in others were crucial for providing a conclusive molecular diagnosis in our borderline cases.

Without the technological stock set up for the molecular tests, 30 patients with borderline methylation levels in our SRS and BWS cohorts would have remained “suspected.” Considering the two opposite growth disorders separately, 9 SRS cases (20 %) and 21 “borderline” BWS cases (more than 10 %) were among the total 43 and 206 molecularly diagnosed cases, respectively.

The fact that the fraction of SRS cases that could be molecularly diagnosed was higher than that of BWS cases could be ascribed to the clinical phenotype, which in general was more complete in SRS and hence promoted the use of genetic testing. This view is exemplified by SRS-1, SRS-5, and SRS-9 cases who all displayed strongly suggestive clinical features, although they obtained molecular diagnosis only by comparative techniques on blood and/or buccal swabs. It is worth to underline that SRS-5 developed a paravertebral neuroblastoma, as tumor occurrence has been very rarely reported in SRS [32].

Apart from this difference, both SRS and BWS borderline cases showed a wide clinical expressivity ranging from overt to incomplete or atypical phenotypes.

A case well exemplifying the challenges of clinical diagnosis and the utility of complementary molecular diagnostic technologies, when a suggestive phenotype is lacking, is SRS-6 who did not display the typical SRS facial dysmorphisms (Fig. 5a) or growth retardation (Table 1) and was referred to our laboratory for hemihyperplasia (i.e., within the BWS spectrum). The molecular diagnosis, at cut-off level by SB and MS-MLPA and then supported by pyrosequencing, allowed revision of the patient’s clinical condition, which was defined as hemihypoplasia (i.e., within the SRS spectrum). The revised diagnosis implied the possibility of waiving the cancer surveillance protocol generally prescribed in IH/BWS cases, which consists of cancer plasma marker measurement and abdominal ultrasound every 3 months during the first 10 years of life. Moreover, this case well represents the seldom encountered “mixed phenotypes” bridging the two opposite growth disorders.

The clinical features of the genetically borderline BWS cases, showing intermediate aberrant methylation values (between mean ±1 SD and mean ±3 SD), were far from the classic phenotypes depicted in cases with a definite molecular diagnosis. Overgrowth at birth, which is usually present in >95 % of BWS patients, was detected in 31 % of borderline cases, and macroglossia, which is the trait described as most indicative of BWS clinical diagnosis, occurred in 33 % of this cohort versus the 70–90 % reported previously [8, 9]. BWS-3 and BWS-7 showed atypical phenotypes and needed other tests, such as array CGH and exome sequencing, to exclude the concurrence of other molecular defects. BWS-7 manifested paroxysmal tonic upgaze, a peculiar sign in BWS patients, and developed persistent hypoglycemia. BWS-3 showed IUGR, postnatal growth retardation up to late infancy, and external hydrocephaly, a sign recently highlighted in a few BWS cases [33]. In this patient, the BWS phenotype was manifested later, when the hemihyperplasia became apparent and Wilms’ tumor developed. Another borderline patient finally diagnosed with *H19/IGF2*:IG-DMR defect, BWS-1, had Wilms’ tumor, and the upd(11)pat carriers BWS-18 and BWS-21 had a benign thyroid and adrenal nodule, respectively.

The widespread occurrence of hemihyperplasia often associated with no or a few other signs supports the observation/hypothesis that a low mosaicism rate, as detected in a few of our upd(11)pat carriers, is likely responsible for the high fraction of IH cases remaining without molecular diagnosis.

As to the wide clinical expressivity of the borderline cases, concomitant factors such as multilocus methylation disturbances (MLID) [34], which may determine a relatively more severe and atypical phenotype than that of only 11p15 epimutated cases, are possibly implicated in borderline BWS cases, impairing (epi)genotype-phenotype correlations [35]. As it has been recently shown, MLID can be exhaustively detected by applying whole genome methylation approaches [36, 37] that although not yet performed in the diagnostic work-up, should be considered as further proceeding as highlighted in our BWS flowchart (Fig.4b) in cases who bear a primary epimutation, including the borderline ones. Another proceeding suitable for BWS cases without molecular diagnosis, especially familial cases, is represented by exome targeted deep sequencing (Fig. 4b) aiming at identifying mutations in genes encoding trans-acting regulators of imprinted loci [38].

The key message of our work is that efforts should be made to solve suspected cases in the SRS and BWS spectrum through various combinations of 11p15.5-targeted and cytogenomic techniques, not only for research purposes but also for the current diagnostic activity.

## Conclusions

MS-MLPA or another eligible technique for the assessment of *H19/IGF2*:IG-DMR and *H19/IGF2*:IG-DMR /*KCNQ1OT1*:TSS-DMR epigenetic alterations may be the starting point of SRS and BWS genetic testing. The MS-MLPA results can orient the molecular geneticist in the application of cytogenomic techniques such as array CGH, SNP array, karyotyping, or targeted FISH to search for other alterations and to detect the slightest mosaicism levels within the limits of the available up-to-date methods. For BWS, besides upd(11)pat, chromosomal rearrangements may be at the basis of simultaneous *H19/IGF2*:IG-DMR and *KCNQ1OT1*:TSS-DMR dysregulation, featuring a rare condition with a recurrence risk of 50 %. Finally, *CDKN1C* mutations should be investigated, not only in all familial cases but also in sporadic cases presenting with omphalocele, both for research and diagnostic purposes.

The diagnostic detection rate could also be improved by introducing the analysis of tissues other than blood into the routine follow-up, as suggested by the occurrence of low-rate mosaicism and the variable degree of methylation in different tissues. The development of more sensitive technical tools should facilitate the identification of cases that may remain undiagnosed because of mosaicism, a distinctive feature of epigenetic disorders.

## Methods

### Subjects

A total of 147 patients aged 6 months to 20 years presenting with IUGR, mild to severe postnatal growth retardation and asymmetry, and facial phenotypes suggestive or SRS, were referred to our laboratory for molecular diagnosis. A sample of 450 patients, ranging from newborns to patients 47 years of age with a clinical diagnosis of BWS, was processed in parallel. The BWS cohort included a subset of 83 patients diagnosed with IH. The criteria for clinical diagnosis of SRS were those established in 2007 by Netchine [39] and recently updated by Azzi (2015) [23] and for BWS, those indicated by Choufani [40]. In all cases, informed consent for genetic testing was signed by the probands or their parents. DNA was extracted from peripheral blood lymphocytes (Automated extractor Tecan, Männedorf, Switzerland and Promega kit, Madison, WI) of the trios of probands and parents; whenever possible, epithelial buccal cells were collected (Oragene tubes OG-575), and DNA was extracted according to the manufacturer’s protocol (Oragene DNA kit, DNA Genotek Inc., a subsidiary of OraSure Technologies).

SRS patients were investigated for upd(7)mat and *H19/IGF2*:IG-DMR LoM and 11p15.5 micro-rearrangements; the BWS cohort and the set of IH cases were tested for UPD11 and *H19/IGF2*:IG-DMR and *KCNQ1OT1*:TSS-DMR defects. In case of uncertain results, a different tissue (epithelial buccal cells) was examined.

The study protocol was approved by the Research Ethics Board of Istituto Auxologico Italiano, Milan, Italy, and all subjects provided written informed consent.

### Uniparental disomy of chromosome 7

A panel of 20 microsatellites, spanning the whole chromosome from pter to qter, D7S517(7p22.2), D7S641(7p21.3), D7S2464(7p21.3), D7S513(7p21.3), D7S507(7p21.1), D7S503(7p21.1), D7S2493(7p15.3), D7S2525(7p15.2), D7S2496(7p14.3), D7S519(7p13), D7S2422(7p12.1), D7S2467(7p12.1), D7S506(7p12.1), D7S1870(7q11.23), D7S486(7q31.2), D7S640(7q32.3), D7S661(7q35), D7S636(7q36.1), D7S798(7q36.2), D7S2465(7q36.3) was selected for segregation analysis from parents to SRS probands. PCR analyses were performed using fluoresceinated primers and PCR products were separated using an automated ABI 310 sequencer.

### Uniparental disomy of chromosome 11

The 11 polymorphic loci (D11S1363, D11S1318, D11S1984, D11S4177, D11S4046, TH, D11S4124, D11S4146, D11S1338, D11S1323, and D11S1760) from 11p15.5 to 11p14 were used for segregation analysis from parents to BWS and IH probands. Mosaicism occurrence and level were assessed by calculating the ratio between maternal and paternal peak areas as reported previously [29]. Additional 11p centromeric markers D11S4116, D11S4121, D11S902, and D11S935 and the 11q markers D11S1777, D11S4191, D11S1883, D11S987, D11S4147, D11S908, D11S4094, and D11S968 were used to establish isodisomy extent in upd carriers.

### *H19* and *KvDMR1* methylation profiles

#### MS-MLPA technique

The MCR-Holland kit, ME-030 BWS/RSS (MRC Holland, Amsterdam, The Netherlands) was used according to the kit instructions on test and control samples (in the ratio of one control for every seven patients). DNA was processed in parallel with and without digestion with the methylation sensitive *HhaI* enzyme to detect both methylation deregulation and copy number variation. Data analysis was performed using the Coffalyser.net software v. 131211, which provides two outputs, one related to CNVs and the other to the methylation status. The latter is defined for each single probe by the ratio of digested to undigested DNA, referring each test sample to positive and negative references.

#### Pyrosequencing

Sodium bisulphite conversion of DNA (500–700 ng) was performed by the EZ DNA Methylation Kit (Zymo Research Corporation, Orange, CA). ICR1, *H19* promoter, *IGF2*-DMR2 (11p15.5 telomeric cluster), and ICR2 (11p15.5 centromeric cluster) PCR analyses were performed on bisulphite-treated DNA using forward and reverse primers, one of which was biotinylated [27]. Pyrosequencing experiments were performed using specific sequencing primers to quantify four CpG sites for ICR1, three for the *H19* promoter, six for DMR2, and four for ICR2. PCR and sequencing primers were as follows:

ICR1 Fw: 5′-TGGGTATTTTTGGAGGTTTTTTT-3′; ICR1 Rev: 5′bio-AACTTAAATCCCAAACCATAACA-3′; ICR1 Seq: 5′-GTTTYGGGTTATTTAAGTTA-3′ (hg19, chr11:2020978-2021291)

H19 promoter Fw: 5′-TGGTGTTTTTTGAGGGGAGATA-3′; Rev: 5′bio-CACCTCCRCCCTAAACAAT-3′; H19 prom Seq: 5′-GGGGTAATGTTTAGTTTTGT-3′ (hg19 , chr11:2019185-2019372)

DMR2 Fw: bio 5′-GGAAGAGYGTGGAGAGTAGGTATTTGTTG-3′; DMR2 Rev: 5′ACTCACTTCCRATTACTAACCATCTC-3′; DMR2 Seq: 5′-CTCRAACTCCTTAACAAAC-3′ (hg19 chr11:2154216-2154503)

ICR2 Fw: 5′-TTGTTTATAAGGTGTAGATGGGAG-3′; Rev: 5′-TCTCCCAAACTCTCCTCAAC-3′; ICR2 Seq: 5′-TAGGTTAGGTTGTATTGTTG-3′ (hg19 chr11:2720465-2720669)

Quantitative DNA methylation analyses were performed using a Pyro Mark ID instrument (QIAGEN, Silicon Valley, CA) in the PSQ HS 96 System with the PyroGold SQA reagent kit (Diatech Pharmacogenetics srl, Jesi, Italy) according to the manufacturer’s instructions. Raw data were analyzed using Q-CpG software v1.0.9 (Qiagen srl), which calculates the ratio of converted C’s (T’s) to unconverted C’s at each CpG, giving the percentage of methylation.

For each sample, the methylation value represents the mean between at least two independent PCR and pyrosequencing experiments. Methylation values were expressed as percentages, and the results were analyzed keeping into account the following ranges of control individuals: ICR1, 40–52 %; *H19* promoter, 44–54 %; DMR2, 41–52 %; and ICR2, 39–50 %.

#### Southern blot analysis

DNA methylation of *H19/IGF2*:IG-DMR (hg19: chr11:2022881-2023267 (left probe), chr11:2020629-2021022 (right probe)) and *KCNQ1OT1*:TSS-DMR (chr11:2722937-2723377) was analyzed by Southern blot hybridization of *H19-*DMR (provided by Prof. A. Riccio, CNR Institute of Genetics and Biophysics, Naples) and *KvDMR1* probes to genomic DNA (7 ug) digested with *Csp6I*/*HpaII* and *BamHI*/*NotI* restriction enzymes, respectively [18]. Filters were washed for 20 min in 2× SSC/0.1 % SDS at room temperature, 15 min in 0.5× SSC/0.1 % SDS at 60 °C and 2 min in 0.1× SSC at room temperature and subject to autoradioradiography. For each single experiment, a negative (unaffected) and a known positive (e.g., a previously tested positive sample) control DNAs were included as internal control references. Autoradiography films were scanned at maximum resolution and signal intensities were quantified using ImageJ software (http://imagej.nih.gov/ij/, version 1.50b). For each lane, the intensities of *H19/IGF2*:IG-DMR (1.7, 1.4, 0.7, 0.5, and 0.4 kb) and *KCNQ1OT1*:TSS-DMR (6 and 1.8 Kb) bands were quantified to calculate methylation index values (MI) according to Lennertz (2010) [41]. A reference methylation index range was obtained by calculating MI of 50 (*H19/IGF2*:IG-DMR) and 40 (*KCNQ1OT1*:TSS-DMR) unaffected individuals. Diagnostic threshold was set as mean value ±2 SD: *H19/IGF2*:IG-DMR (SRS LoM <44; BWS GoM >60), *KCNQ1OT1*:TSS-DMR (BWS LoM <43).

#### SNP array

SNP array analysis was performed using the Human OmniExpress-12 Bead Chip (Illumina Inc., San Diego, CA) containing 731,442 loci derived from phases I, II, and III of the International HapMap project. A total of 200 ng of genomic DNA (50 ng/μl) for each sample was processed according to Illumina’s Infinium HD Assay protocol. Normalization of raw image intensity data, genotype clustering, and individual sample genotype calls were performed using Illumina’s GenomeStudio software v2011.1 (cnvpartition 3.1.6). The CNV calls were determined using generalized genotyping methods implemented in the PennCNV program. The CNVs were mapped to the human reference genome hg19 and annotated with the UCSC RefGene. CNVs identified in this study that overlapped with CNVs reported in the Database of Genomic Variants (DGV) (http://projects.tcag.ca/variation) were not considered.

## Additional files

Additional file 1: Table S1. Normal methylation ranges and thresholds calculated on different sets of healthy individuals by Southern blot, MS-MLPA, and pyrosequencing. A) Southern blot methylation thresholds calculated by densitometry on a set of 50 and 40 Italian healthy controls for *H19/IGF2*:IG-DMR and *KCNQ1OT1*:TSS-DMR, respectively, B) MS-MLPA range of methylation ratios defined on a set of 50 Italian healthy controls, and C) pyrosequencing methylation range (in percentage) obtained by control individuals. For details, see the “Methods” section. (PDF 14 kb) Additional file 2: Table S2. SRS and BWS patients who achieved molecular diagnosis by different techniques. Column 2 indicates the number of patients for SRS and BWS, who received a positive molecular diagnosis, apart from *CDKN1C* mutated patients; column 4 reports the number of patients with a definite positive test and a borderline test. In column 5, the investigated defect is reported, and in column 6, the number of patients for each mechanism. The number of cases tested for SB, MS-MLPA, four pyrosequencing probes, microsatellite analysis, and SNP array are reported in the following columns. Some cases with definite result were replicated by MS-MLPA and pyrosequencing for their set up. The last column indicates the cases submitted to SNP array to characterize chromosomal rearrangements (rows 5 and 6) or to disclose low mosaicism for upd(11)pat (row 9). The table does not refers to the outcome of the test.*Numbers include three *H19/IGF2*:IG-DMR GoM and four *H19/IGF2*:IG-DMR GoM + *KCNQ1OT1*:TSS-DMR LoM cases who needed karyotyping /FISH analysis, three of which reported [18, 28]. (PDF 38 kb) Additional file 3: Table S3. Comparison of methylation data obtained by Southern blot, pyrosequencing, and MS-MLPA of all borderline (BL) SRS and BWS cases. Methylation indexes (MI) and values obtained by SB, pyrosequencing, and MS-MLPA techniques of (A) SRS borderline LoM (*H19/IGF2*:IG-DMR), (B) BWS borderline GoM (*H19/IGF2*:IG-DMR), (C) BWS borderline LoM (*KCNQ1OT1*:TSS-DMR), (D) BWS borderline upd GoM (*H19/IGF2*:IG-DMR), (E) BWS borderline upd LoM (*KCNQ1OT1*:TSS-DMR), and (F) SRS borderline case 1 (blood vs buccal swab) (*H19/IGF2*:IG-DMR). BWS borderline cases (12, 13, 14, 15, 16, and 21) also investigated by SNP array. Methylation data of all techniques were calculated as described in the “Methods” section. Missing data are indicated as nd (not detected). Asterisks (*) indicate the mean of at least two experiments. Diagnostic thresholds (see text, the “Methods” and Additional file 1 for exhaustive explanations) are shown on the right side. MS-MLPA thresholds obtained by adding or subtracting (to the average value) three (in black) or two (in red) standard deviations are also displayed. Aberrant methylation values are bolded and underlined while methylation values meeting less stringent criteria (±2 SD) are in red characters. (PDF 57 kb)
