# Erratum to: A multi-method approach to the molecular diagnosis of overt and borderline 11p15.5 defects underlying Silver–Russell and Beckwith–Wiedemann syndromes

**DOI:** 10.1186/s13148-016-0206-5

**Published:** 2016-04-21

**Authors:** Silvia Russo, Luciano Calzari, Alessandro Mussa, Ester Mainini, Matteo Cassina, Stefania Di Candia, Maurizio Clementi, Sara Guzzetti, Silvia Tabano, Monica Miozzo, Silvia Sirchia, Palma Finelli, Paolo Prontera, Silvia Maitz, Giovanni Sorge, Annalisa Calcagno, Mohamad Maghnie, Maria Teresa Divizia, Daniela Melis, Emanuela Manfredini, Giovanni Battista Ferrero, Vanna Pecile, Lidia Larizza

**Affiliations:** Human Molecular Genetics Laboratory, IRCCS Istituto Auxologico Italiano, Milano, Italy; Department of Pediatric and Public Health Sciences, University of Turin, Torino, Italy; Clinical Genetics Unit, Department of Women’s and Children’s Health, University of Padua, Padova, Italy; Department of Pediatrics, San Raffaele Scientific Institute, Milano, Italy; Division of Pathology - Fondazione IRCCS Ca’ Granda Ospedale Maggiore Policlinico, Department of Pathophysiology and Transplantation, University of Milan, Milano, Italy; Department of Health Sciences, University of Milan, Milano, Italy; Medical Genetics Unit, Department of Surgical and Biomedical Sciences, University of Perugia, Hospital “S. M. della Misericordia”, Perugia, Italy; Clinical Pediatric Genetics Unit, Pediatrics Clinics, MBBM Foundation, S. Gerardo Hospital, Monza, Italy; Department of Pediatrics and Medical Sciences, AO “Policlinico Vittorio Emanuele”, Catania, Italy; Pediatric Endocrine Unit, Department of Pediatrics, IRCCS, Children’s Hospital Giannina Gaslini, Genova, Italy; Department of Medical Genetics, IRCCS, Children’s Hospital Giannina Gaslini, Genova, Italy; Clinical Pediatric Genetics, Department of Pediatrics, University “Federico II”, Napoli, Italy; Medical Genetics Unit, Department of Laboratory Medicine, Niguarda Ca’ Granda Hospital, Milano, Italy; Institute for Maternal and Child Health, Foundation IRCCS Burlo Garofolo Institute, Trieste, Italy; Department of Neuroscience, Rehabilitation, Ophtalmology, Genetics, Maternal and Child Health, University of Genova, Genova, Italy

## Erratum

Unfortunately, the original version of this article [1] contained two errors. Within the “Acknowledgements” section, the funding information was missing, and the second affiliation for Mohamad Maghnie was missing. The correct author list and “Acknowledgements” section are included in this erratum.
