# Supplementary material for: A multi-method approach to the molecular diagnosis of overt and borderline 11p15.5 defects underlying Silver–Russell and Beckwith–Wiedemann syndromes
Source: Clin Epigenetics. 2016 Mar 1;8:23. doi: 10.1186/s13148-016-0183-8 (PMC4772365; doi:10.1186/s13148-016-0183-8)
Supplement: Additional file 1: Table S1. — Normal methylation ranges and thresholds calculated on different sets of healthy individuals by Southern blot, MS-MLPA, and pyrosequencing. A) Southern blot methylation thresholds calculated by densitometry on a set of 50 and 40 Italian healthy controls for H19/IGF2:IG-DMR and KCNQ1OT1:TSS-DMR, respectively, B) MS-MLPA range of methylation ratios defined on a set of 50 Italian healthy controls, and C) pyrosequencing methylation range (in percentage) obtained by control individuals. For details, see the “Methods” section. (PDF 14 kb) [file 13148_2016_183_MOESM1_ESM.pdf]

Additional file 1. Normal methylation ranges and thresholds calculated on different sets of healthy individuals by Southern blot, MS-MLPA and pyrosequencing.

A) Southern blot.

	***H19/IGF2*:IG-DMR MI (n=50)**
**Mean**	**52**
**Standard Dev.**	4
**+2 SD**	60
**- 2 SD**	44

	***KCNQ1OT1*:TSS-DMR MI (n=40)**
**Mean**	**51**
**Standard Dev.**	4
**- 2 SD**	43

B) MS-MLPA.

	***H19/IGF2*:IG-DMR *HhaI* probes**					***KCNQ1OT1*:TSS-DMR *HhaI* probes**				
**Controls (50)**	**(1) L05772**	**(2) L16503**	**(3) L08764**	**(4) L20532**	**ALL PROBES**	**(1) L19204**	**(2) L05782**	**(3) L06781**	**(4) L19191**	**ALL PROBES**
**Mean**	**0.48**	**0.51**	**0.52**	**0.55**	**0.52**	**0.53**	**0.49**	**0.51**	**0.56**	**0.52**
**Standard Dev.**	0.02	0.04	0.03	0.04	0.03	0.03	0.02	0.03	0.04	0.03
**Max value**	0.52	0.58	0.58	0.61		0.59	0.54	0.57	0.63	
**Min value**	0.44	0.43	0.46	0.48		0.48	0.45	0.44	0.49	
**+3 SD**	0.54	0.63	0.61	0.67	0.61	0.61	0.56	0.60	0.69	0.61
**+2 SD**	0.52	0.59	0.58	0.63	0.58	0.59	0.54	0.57	0.65	0.58
**+1 SD**	0.50	0.55	0.55	0.59	0.55	0.56	0.52	0.54	0.60	0.55
**Mean**	**0.48**	**0.51**	**0.52**	**0.55**	**0.52**	**0.53**	**0.49**	**0.51**	**0.56**	**0.52**
**-1 SD**	0.46	0.47	0.49	0.51	0.49	0.50	0.47	0.48	0.52	0.49
**-2 SD**	0.44	0.43	0.46	0.47	0.46	0.47	0.45	0.45	0.48	0.46
**-3 SD**	0.42	0.39	0.43	0.43	0.43	0.44	0.43	0.42	0.44	0.43

C) Pyrosequencing.

ICR1: 40–52%

***H19* promoter**: 44–54%;

DMR2: 41–52%

ICR2: 39–50%.
