# Supplementary material for: A multi-method approach to the molecular diagnosis of overt and borderline 11p15.5 defects underlying Silver–Russell and Beckwith–Wiedemann syndromes
Source: Clin Epigenetics. 2016 Mar 1;8:23. doi: 10.1186/s13148-016-0183-8 (PMC4772365; doi:10.1186/s13148-016-0183-8)
Supplement: Additional file 2: Table S2. — SRS and BWS patients who achieved molecular diagnosis by different techniques. Column 2 indicates the number of patients for SRS and BWS, who received a positive molecular diagnosis, apart from CDKN1C mutated patients; column 4 reports the number of patients with a definite positive test and a borderline test. In column 5, the investigated defect is reported, and in column 6, the number of patients for each mechanism. The number of cases tested for SB, MS-MLPA, four pyrosequencing probes, microsatellite analysis, and SNP array are reported in the following columns. Some cases with definite result were replicated by MS-MLPA and pyrosequencing for their set up. The last column indicates the cases submitted to SNP array to characterize chromosomal rearrangements (rows 5 and 6) or to disclose low mosaicism for upd(11)pat (row 9). The table does not refers to the outcome of the test.*Numbers include three H19/IGF2:IG-DMR GoM and four H19/IGF2:IG-DMR GoM + KCNQ1OT1:TSS-DMR LoM cases who needed karyotyping /FISH analysis, three of which reported [18, 28]. (PDF 38 kb) [file 13148_2016_183_MOESM2_ESM.pdf]

Additional file 2. SRS and BWS patients who achieved molecular diagnosis by different techniques.

Patients with diagnosis	N°	Diagnosis	N°	Mechanism	N°	Southern blot	MS-MLPA	Pyrosequencing				Microsatellite analysis	SNP-array
								ICR1	*H19* prom	*IGF2* -DMR2	ICR2		
**SRS**	**43**	**definite**	**34**	***H19 /IGF2*** **:IG-DMR LOM**	**27**	25	23	26	10	0	0	13	0
				**UPD7**	**7**	7	4					7	0
		**borderline**	**9**	***H19/IGF2*** **:IG-DMR LOM**	**9**	9	9	6	0	0	0	9	0
**BWS**	**196**	**definite**	**175**	***KCNQ1OT1*** **:TSS-DMR LOM**	**120**	102	48	2	0	0	2	91	0
				H19 /IGF2 **:IG-DMR GOM**	**9***	9	6	4	4	0	4	9	3
				KCNQ1OT1 **:TSS-DMR LOM and** ***H19/IGF2*** **:IG-DMR GOM**	**46***	38	18	3	2	0	3	45	4
		**borderline**	**21**	***KCNQ1OT1*** **:TSS-DMR LOM**	**8**	7	8	8	0	0	7	4	0
				H19 /IGF2 **:IG-DMR GOM**	**3**	3	3	3	3	3	3	3	0
				**UPD11**	**10**	8	8	10	4	0	10	10	6
