# Supplementary material for: A multi-method approach to the molecular diagnosis of overt and borderline 11p15.5 defects underlying Silver–Russell and Beckwith–Wiedemann syndromes
Source: Clin Epigenetics. 2016 Mar 1;8:23. doi: 10.1186/s13148-016-0183-8 (PMC4772365; doi:10.1186/s13148-016-0183-8)
Supplement: Additional file 3: Table S3. — Comparison of methylation data obtained by Southern blot, pyrosequencing, and MS-MLPA of all borderline (BL) SRS and BWS cases. Methylation indexes (MI) and values obtained by SB, pyrosequencing, and MS-MLPA techniques of (A) SRS borderline LoM (H19/IGF2:IG-DMR), (B) BWS borderline GoM (H19/IGF2:IG-DMR), (C) BWS borderline LoM (KCNQ1OT1:TSS-DMR), (D) BWS borderline upd GoM (H19/IGF2:IG-DMR), (E) BWS borderline upd LoM (KCNQ1OT1:TSS-DMR), and (F) SRS borderline case 1 (blood vs buccal swab) (H19/IGF2:IG-DMR). BWS borderline cases (12, 13, 14, 15, 16, and 21) also investigated by SNP array. Methylation data of all techniques were calculated as described in the “Methods” section. Missing data are indicated as nd (not detected). Asterisks (*) indicate the mean of at least two experiments. Diagnostic thresholds (see text, the “Methods” and Additional file 1 for exhaustive explanations) are shown on the right side. MS-MLPA thresholds obtained by adding or subtracting (to the average value) three (in black) or two (in red) standard deviations are also displayed. Aberrant methylation values are bolded and underlined while methylation values meeting less stringent criteria (±2 SD) are in red characters. (PDF 57 kb) [file 13148_2016_183_MOESM3_ESM.pdf]

Additional File 3. Comparison of methylation data obtained by Southern blot, pyrosequencing and MS-MLPA of all borderline (BL) SRS and BWS cases.

A

	H19 /IGF2 :IG-DMR										
	SRS BL LoM										
	1	2	3	4	5	6	7	8	9	*Threshold*	
SB MI	37	36	45	42	26	43	39	39	50	*< 44*	
Pyroseq. ICR1	37	41	37	37	nd	36	nd	nd	40	*< 40*	
MS-MLPA	RATIO*	RATIO*	RATIO*	RATIO	RATIO	RATIO	RATIO*	RATIO*	RATIO*	*- 3 sd*	*- 2 sd*
(1) L05772	0.40	0.40	0.40	0.31	0.33	0.31	0.35	0.34	0.38	*≤ 0.42*	*≤ 0.44*
(2) L16503	0.37	0.39	0.37	0.33	0.33	0.32	0.37	0.34	0.35	*≤ 0.39*	*≤ 0.43*
(3) L08764	0.42	0.41	0.40	0.40	0.34	0.42	0.38	0.40	0.46	*≤ 0.43*	*≤ 0.46*
(4) L20532	0.40	0.46	0.46	0.42	0.36	0.36	0.44	0.43	0.46	*≤ 0.43*	*≤ 0.47*
mean of MSMLPA values	0.40	0.42	0.41	0.37	0.34	0.35	0.38	0.38	0.41	*≤ 0.43*	*≤ 0.46*

B

	H19 /IGF2 :IG-DMR				
	BWS BL GoM				
	1	2	3	*Threshold*	
SB MI	61	57	63	*> 60*	
Pyroseq. ICR1	44	48	nd	*> 52*	
Pyroseq. H19 prom	49	56	55	*> 54*	
Pyroseq. DMR2	56	51	nd	*> 52*	
MS-MLPA	RATIO*	RATIO*	RATIO*	*+ 2 sd*	*+ 3 sd*
(1) L05772	0.52	0.58	0.58	*≥ 0.52*	*≥ 0.54*
(2) L16503	0.58	0.60	0.64	*≥ 0.59*	*≥ 0.63*
(3) L08764	0.58	0.63	0.71	*≥ 0.58*	*≥ 0.61*
(4) L20532	0.69	0.7	0.64	*≥ 0.63*	*≥ 0.67*
mean of MSMLPA values	0.59	0.63	0.64	*≥ 0.58*	*≥ 0.61*

C

	KCNQ1OT1 :TSS-DMR									
	BWS BL LoM									
	4	5	6	7	8	9	10	11	*Threshold*	
SB MI	46	37	43	25	29	39	31	nd	*< 43*	
Pyroseq. ICR2	38	31	37	36	30	37	43	38	*< 39*	
MS-MLPA	RATIO*	RATIO	RATIO*	RATIO*	RATIO	RATIO*	RATIO*	RATIO*	*- 3sd*	*- 2sd*
(1) L19204	0.40	0.35	0.42	0.39	0.35	0.38	0.51	0.45	*≤ 0.44*	*≤ 0.47*
(2) L05782	0.37	0.31	0.39	0.36	0.29	0.36	0.49	0.41	*≤ 0.43*	*≤ 0.45*
(3) L06781	0.41	0.33	0.38	0.37	0.33	0.38	0.45	0.43	*≤ 0.42*	*≤ 0.45*
(4) L19191	0.44	0.38	0.53	0.37	0.33	0.39	0.46	0.44	*≤ 0.44*	*≤ 0.48*
mean of MSMLPA values	0.41	0.34	0.43	0.37	0.33	0.38	0.48	0.43	*≤ 0.43*	*≤ 0.46*

D

	H19 /IGF2 :IG-DMR											
	BWS BL UPD GoM											
	12	13	14	15	16	17	18	19	20	21	*Threshold*	
SB MI	60	65	51	66	63	64	52	70	66	59	*> 60*	
Pyroseq. ICR1	46	49	47	44	46	47	47	45	47	44	*> 52*	
Pyroseq. H19 prom			55	61		56	54.5				*> 54*	
MS-MLPA	RATIO*	RATIO*	RATIO*	RATIO*	RATIO*	RATIO*	RATIO	RATIO*	RATIO	RATIO*	*+ 2 sd*	*+ 3 sd*
(1) L05772	0.61	0.52	0.55	0.58	0.54	0.62	0.61	0.64	0.61	0.55	*≥ 0.52*	*≥ 0.54*
(2) L16503	0.59	0.54	0.53	0.61	0.57	0.64	0.59	0.58	0.66	0.65	*≥ 0.59*	*≥ 0.63*
(3) L08764	0.66	0.57	0.59	0.59	0.61	0.62	0.58	0.63	0.65	0.62	*≥ 0.58*	*≥ 0.61*
(4) L20532	0.70	0.62	0.63	0.70	0.66	0.66	0.62	0.66	0.61	0.66	*≥ 0.63*	*≥ 0.67*
mean of MSMLPA values	0.64	0.56	0.58	0.62	0.60	0.64	0.60	0.63	0.63	0.62	*≥ 0.58*	*≥ 0.61*

E

	KCNQ1OT1 :TSS-DMR											
	BWS BL UPD LoM											
	**12**	**13**	**14**	**15**	**16**	**17**	**18**	**19**	**20**	**21**	***Threshold***	
**SB MI**	46	47	**30**	43	**41**	43	52	**34**	**42**	**42**	***< 43***	
**Pyroseq. ICR2**	**38**	41	**38**	43	43	**36**	**37**	**35**	42	**37**	***< 39***	
**MS-MLPA**	RATIO*	RATIO*	RATIO*	RATIO*	RATIO*	RATIO*	RATIO	RATIO*	RATIO	RATIO*	***- 3sd***	*- 2sd*
**(1) L19204**	**0.44**	0.49	0.45	0.46	0.49	0.48	**0.43**	**0.41**	**0.40**	0.48	***≤ 0.44***	*≤ 0.47*
**(2) L05782**	0.50	0.47	**0.42**	**0.43**	0.46	**0.42**	**0.41**	**0.37**	**0.39**	**0.42**	***≤ 0.43***	*≤ 0.45*
**(3) L06781**	**0.41**	0.48	0.44	0.44	0.48	0.47	0.44	**0.37**	**0.41**	0.45	***≤ 0.42***	*≤ 0.45*
**(4) L19191**	0.51	0.48	0.49	0.47	0.48	**0.44**	**0.44**	0.47	**0.43**	**0.44**	***≤ 0.44***	*≤ 0.48*
**mean of MSMLPA values**	0.46	0.48	0.45	0.45	0.48	0.45	**0.43**	**0.41**	**0.41**	0.45	***≤ 0.43***	*≤ 0.46*

F

	H19 /IGF2 :IG-DMR			
	**Blood**	**Buccal Swab**		
	**SRS-1**	**SRS-1**	***Threshold***	
**SB MI**	**37**	**36**	***< 44***	
**Pyroseq. ICR1**	**37**	**28**	***< 40***	
**MS-MLPA**	RATIO*	RATIO*	***- 3sd***	*- 2sd*
**(1) L05772**	**0.40**	**0.37**	***≤ 0.42***	*≤ 0.44*
**(2) L16503**	**0.37**	**0.32**	***≤ 0.39***	*≤ 0.43*
**(3) L08764**	**0.42**	**0.39**	***≤ 0.43***	*≤ 0.46*
**(4) L20532**	**0.40**	**0.43**	***≤ 0.43***	*≤ 0.47*
**mean of MSMLPA values**	**0.40**	**0.38**	***≤ 0.43***	*≤ 0.46*
